# Fellowships, Grants, & Awards

**Published:** 2006-09

## NIDDK Career Transition Award (K22) in Patient-Oriented Research

The overall aim of the National Institute of Diabetes and Digestive and Kidney Diseases (NIDDK) Career Transition Award (K22) is to enable outstanding physician-scientists to obtain clinical research training experiences in the NIDDK Intramural Research Program and to facilitate their successful transition to an extramural environment as independent researchers. The award will provide up to 3 years of support for research training in an NIDDK intramural clinical laboratory followed by 2 years of support for an independent research program at an extramural institution. The combined duration may not exceed 5 years. It is anticipated that awardees will subsequently obtain research project grants, such as the R01, to support the continuation of their research.

### NIDDK Intramural Research Program

The NIDDK intramural research environment provides a rich and unique opportunity for the training of postdoctoral fellows and early career scientists. The Intramural Research Program at the NIDDK conducts basic biomedical, translational, and clinical research in the broad areas of diabetes, endocrinology, metabolism, transplantation, hepatitis, digestive and kidney diseases, hematopoiesis, and hemoglobinopathies.

Eligibility for and receipt of this award is dependent upon participation in the NIDDK Senior Clinical Research Fellowship Program (http://fellowshipoffice.niddk.nih.gov/scrfp/). The 3-year program is built on a solid foundation of the NIH–Duke University Clinical Research Program that offers didactic courses in research design and management, statistical analysis, and bioethics. Enrollment in the NIH–Duke University Program is required of all participants in the NIDDK Senior Clinical Research Fellowship Program. There is also direct, hands-on practical experience in clinical investigation through an individualized research project as part of the thesis requirement. The combined course of study ultimately leads to the awarding of a Masters Degree in Clinical Research.

Detailed information about programmatic areas in clinical, translational, and patient-oriented research as well as potential intramural NIDDK mentors, clinical protocols, and laboratories may be obtained at http://fellowshipoffice.niddk.nih.gov/scrfp/ and http://intramural.niddk.nih.gov.

This Program Announcement will use the National Institutes of Health (NIH) Career Transition Award (K22) mechanism. Responsibility for the planning, direction, and execution of the proposed project will reside with the applicant and the proposed mentor (co-mentor).

The total period of support is a maximum of 5 years (3 years of intramural funding and 2 years of extramural funding). Awards are not renewable. Total direct costs for Phase I (intramural) are based on the candidate’s experience; those for Phase II (extramural) may not exceed $175,000 per year (for salary and research support) plus fringe benefits (specific allowable costs are described below).

Transition from Phase I (the intramural period of support) to Phase II (the extramural period) is not automatic. Approval of the transition will be based on the success of the awardee’s research program as determined by yearly scientific review and by the evaluation of a research and career development plan to be carried out at the extramural institution. Additionally, the extramural institution must demonstrate a commitment to the candidate by providing a minimum of 75% protected research time, space, and resources needed to perform the proposed research project and appropriate scientific mentoring. A plan for career development that will lead to research independence and the ability to successfully compete for research support is essential. Awardees approved to proceed with the second phase of support will receive notification of approval in writing from the NIDDK. An application from the extramural institution on behalf of the candidate will be required for the NIDDK to process the second phase of the award (details appear in the section “Activating the Extramural Support Phase” in the online announcement).

This funding opportunity uses just-in-time concepts. The PHS 398 application instructions are available at http://grants.nih.gov/grants/funding/phs398/phs398.html in an interactive format. Applicants must use the currently approved version of the PHS 398. For further assistance contact GrantsInfo, by phone 301-435-0714 (telecommunications for the hearing impaired: TTY 301-451-0088), or by e-mail: GrantsInfo@nih.gov.

Applications must be prepared using the most current PHS 398 research grant application instructions and forms. Applications must have a Dun & Bradstreet Data Universal Numbering System (DUNS) number as the universal identifier when applying for federal grants or cooperative agreements. The D&B number can be obtained by calling 866-705-5711 or through the web site at http://www.dnb.com/us/. The D&B number should be entered on line 11 of the face page of the PHS 398 form.

The application submission dates for this PAR are available at http://grants.nih.gov/grants/funding/submissionschedule.htm. The complete version of this PAR is available at http://grants.nih.gov/grants/guide/par-files/PAR-06-002.

Contacts: Direct questions about the intramural phase, including the initial application process and opportunities for scientific research within the intramural program to: Louis Simchowitz, Office of Fellow Recruitment and Career Development, Division of Intramural Research, National Institute of Diabetes and Digestive and Kidney Diseases, Building 12A, Room 3011, 12 South Drive, Bethesda, MD 20892-5632 USA, 301-451-9808, fax: 301-402-7461, e-mail: LS347F@nih.gov.

Direct questions about the extramural phase to: Judith Podskalny, Division of Digestive Diseases and Nutrition, National Institute of Diabetes and Digestive and Kidney Diseases, 6707 Democracy Boulevard, Room 667, Bethesda, MD 20892-5450 USA, 301-594-8876, fax: 301-480-8300, e-mail: JP52S@nih.gov; Mary Frances Picciano, Office of Dietary Supplements, National Institutes of Health, 6100 Executive Boulevard, Suite 3B01, Bethesda, MD 20892-7517 USA, 301-435-3608, fax: 301-480-1845, e-mail: MP288D@nih.gov.

Reference: PAR-06-002.

## Career Development Program in the Genetics and Genomics of Lung Diseases (K12)

Lung diseases are genetically complex diseases in which many genes and many environmental factors interact. Identification of gene variants associated with such diseases will greatly facilitate the characterization of the contribution that genes and environment play in the development of pathological conditions like asthma and chronic obstructive lung disease. We recognize that, in the pulmonary community, genetic and genomic approaches are not sufficiently well utilized, and this deficiency has limited pulmonary investigators in taking full advantage of the tools and information that have been generated by the human genome project. We believe that this is due in part to the lack of a critical mass of junior investigators properly trained and interested in applying their knowledge to the diseases of the lung.

This new program will utilize the K12 NIH Institutional Mentored Research Scientist Development Program to give M.D. and Ph.D. scientists, who are already committed to the study of lung diseases and who have completed their M.D. or Ph.D. degree within the last 5 years, opportunities to obtain additional career development and training in the genetic bases and genomic analysis of such diseases. They will accomplish this by being part of interdisciplinary research groups in which several different genetic and genomic techniques are being applied to the study of lung diseases.

We encourage applications that propose, at least in part, a specific lung disease–related research theme where each scholar/faculties team would be in charge of a different aspect of the research, such as clinical phenotyping, human sample selection, genotyping and SNPs analysis, expression profile analysis, and the selection and application of the right software for data analysis. This approach to career development would give each scholar an appreciation of the complexity of the issues surrounding a complex lung disease and the need for integration and interaction of the various components.

K12 Institutional Mentored Research Scientist development programs are awards to organizations which include domestic, for-profit and nonprofit, public and private institutions, such as universities, colleges, and hospitals to support career development experiences for new investigators leading to research independence. Under this award, newly trained investigators are to be selected and appointed to this program by the grantee institution.

Individuals who wish to apply as principal investigators (PIs) should be senior-funded investigators who are well established in their areas of research and who will recruit and mentor new scholars. The participating institutions will be encouraged to also develop a formal training curriculum that includes course and hands-on activities.

The primary goal of this RFA is to promote comprehensive genetic and genomic research career development activities, including training for senior postdoctoral fellows and junior faculty–level health professionals who want to pursue academic careers in pulmonary medicine. Through this initiative, the National Heart, Lung, and Blood Institute (NHLBI) will support several multidisciplinary career development programs to prepare these individuals for academic research careers in the genetics and genomics of pulmonary diseases.

The specific research career development objectives of this RFA, which is focused on the areas of genetics and genomics of lung diseases are to: 1) increase national capacity for genetic and genomic research in the area of lung diseases; 2) increase the number of mentors in this area; and 3) equip new academic researchers with the knowledge and skills necessary to address the genetic bases of complex lung diseases.

These objectives stem from the fact that, despite very substantial progress made in the area of genetics and genomics in the last 10 years, the lung research community has been lagging behind other organ-focused research communities in the ability to take advantage of the tools and information generated by the human genome project and other related programs that followed it.

It is important to note that the proposed programs are distinct from traditional clinical fellowships or career development programs. Programs funded by this NHLBI initiative require a 1-year multidisciplinary core curriculum and didactics, and ≥ 1 year of mentored research to foster careers in genetics and genomics of lung diseases.

Through this program, NHLBI will provide awards to organizations that include domestic, for-profit and nonprofit, public and private institutions, such as universities, colleges, and hospitals, in order to develop a core curriculum and a didactic component, and provide the participating researchers with an opportunity to conduct a mentored research project. With this award, scholars should develop the skills, knowledge, and experience to pursue additional grants (e.g., K08, K23, R01), and to become independent investigators and leaders in academic research programs in genetics and genomics of lung diseases. Under the current policy, recipients of K12 support may subsequently apply for NHLBI-supported individual mentored K-series awards (K01, K08, K23) provided that they had > 3 years of K12 support by the time the individual K-series award is issued. The combined total of K12 support plus individual mentored K-series support must not exceed 6 years. Therefore, if an investigator has been supported by a K12 for 3 years, he/she will be able to receive > 3 years of mentored individual career award (K01, K08, K23, etc.).

Programs should emphasize the multifaceted nature of the required career development program which includes training.

The program director (PD) should draw on the strengths of his/her institution, its faculty, and resources to develop a specialized core curriculum in genetics and genomics of lung diseases. The program director, as the scientific and administrative leader of the program, should obtain guidance from a multidisciplinary advisory committee responsible for program oversight and evaluation.

Faculty with expertise in multiple specialties should serve as mentors. They should be responsible for: 1) supervising and evaluating scholars’ research skills development and educational activities; 2) identifying gaps in scholars’ knowledge and skills and formulating plans to fill those gaps; 3) assuring that scholars design and implement a short-term research project that will generate publishable results and preliminary data for a subsequent K08, K23, R01, and/or similar grant application(s); and 4) developing, updating, enhancing, evaluating, and revising curricula and innovative teaching methods and tools.

The core curriculum for the first year should provide: 1) a broad base knowledge of theories, quantitative methods, applications of biotechnology and bioinformatics pertinent to genome analysis; and 2) an introduction to the associated social and ethical implications of development in biotechnology and genomics.

Didactics and hands-on activities in genetics and genomics should expose scholars to: 1) experience in automatic DNA sequencing; 2) high-throughput enzyme assays; 3) high-throughput gene amplification; 4) bioinformatics analyses; 5) DNA microarray construction and analysis; 6) biostatistics; 7) observational and experimental research designs and methods; 8) hypothesis development; 9) responsible conduct of research, and research ethics with human subjects; and 10) presentation and publication of research results.

Scholars entering the program should have completed their M.D. or Ph.D degree within the past 5 years and be interested in applying genetic and genomic techniques to lung diseases. Some individuals may not need to participate in the entire core curriculum or didactics. These scholars may elect additional career development and training in areas of interest, such as bioinformatics, pharmacogenomics, epidemiologic methods, advanced biostatistics, and proteomics. Applicants are encouraged to submit plans to accommodate these potential scholars and to individualize their career development and training experiences.

Late in year 1 and early in year 2, scholars, with guidance and input from both mentors, should design a research project that can be implemented and the data analyzed in the remaining period of support. Applications that focus mainly on lung cancer will not be considered responsive to this RFA.

The applicant institution and affiliated organizations should: 1) have (involved) personnel with the necessary expertise and experience in the conduct of genetics and genomics research; 2) have a strong program of research on pulmonary diseases; 3) have facilities for genomic research; and 4) assure protected time for the program director, mentors, and scholars.

This funding opportunity will use the NIH Institutional Mentored Research Scientist Development Program (K12) grant award mechanism. As an applicant, you will be solely responsible for planning, directing, and executing the proposed project.

This funding opportunity uses the just-in-time budget concepts. It also uses the nonmodular budget format described in the PHS 398 application instructions (see http://grants.nih.gov/grants/funding/phs398/phs398.html). A detailed categorical budget for the Initial Budget Period and the Entire Proposed Period of Support is to be submitted with the application.

The PHS 398 application instructions are available at http://grants.nih.gov/grants/funding/phs398/phs398.html in an interactive format. Applicants must use the currently approved version of the PHS 398. For further assistance, contact GrantsInfo; 301-435-0714 (telecommunications for the hearing impaired: TTY 301-451-0088), or by e-mail: GrantsInfo@nih.gov.

Applications must be prepared using the most current PHS 398 research grant application instructions and forms. Applications must have a D&B Data Universal Numbering System (DUNS) number as the universal identifier when applying for federal grants or cooperative agreements. The D&B number can be obtained by calling 866-705-5711 or through the web site at http://www.dnb.com/us/. The D&B number should be entered on line 11 of the face page of the PHS 398 form.

The letter of intent receipt date for this RFA is 30 October 2006, with the application receipt date 30 November 2006. The complete version of this RFA is available at http://grants.nih.gov/grants/guide/rfa-files/RFA-HL-07-004.html.

Contact: Sandra Colombini Hatch, Lung Biology and Disease Program, Division of Lung Diseases, National Heart, Lung, and Blood Institute, 6701 Rockledge Drive, Rockledge ll Suite 10124, MSC 7952, Bethesda, MD 20892-7952 USA (for U.S. Postal Service express or regular mail) Bethesda, MD 20817 USA (for express/courier delivery; non-USPS service), 301-435-0222, fax: 301480-3557, e-mail: hatchs@nhlbi.nih.gov.

Reference RFA-HL-07-004.

## Robert Wood Johnson Foundation Invites Disparities Research Proposals

The Robert Wood Johnson Foundation (RWJF) seeks to reduce racial and ethnic disparities in the care of patients with cardiovascular disease, diabetes mellitus type 2, and/or depression. To that end, RWJF invites research proposals that offer solutions towards reducing health care disparities.

RWJF encourages researchers to include data and analyses in their proposals to help better understand these disparities related to: 1) subethnic and other marginalized groups (e.g., Mexican, Puerto Rican, Vietnamese, and American Indian); and 2) acculturation factors (e.g., generation, years in United States, age of migration, and language proficiency).

They will consider projects of up to $75,000 with a 1-year time frame that address one or more of five key issues: 1) What is known about the quality of care provided by the hospitals, physicians, and community clinics that deliver the majority of care to diverse patient populations? 2) Is health information technology (HIT) a promising intervention for reducing racial and ethnic disparities in health care? 3) What differences in quality of care exist for Hispanic/Latino subethnic groups (including Puerto Rican, Mexican, Central or South American, and others) and Asian subethnic groups (including Filipino, Vietnamese, Cambodian, Chinese, and others)? We are also interested in studies that focus on American Indians, Alaska Natives, Native Hawaiians and subgroups within the black or African-American population (Caribbean, African, West Indian, or other islander. 4) While some researchers continue to document disparities in care, others have found decreased, declining or a complete absence of racial and ethnic disparities in some types of quality of care indicators. How do these findings fit into the context of the wider disparities literature? 5) How does patient-centered care relate to better clinical outcomes?

Investigators must have at least 5 years of postdoctoral research experience. These projects are not intended for doctoral students or recent graduates.

When submitting a brief proposal, please be sure to include the phrase "Disparities research questions" in the project title.

For more information, visit these websites: http://www.rwjf.org/global/email.jsp?id=677; http://www.rwjf.org/portfolios/features/featuredetail.jsp?featureID=1586=3=133; http://www.rwjf.org/portfolios/features/featuredetail.jsp?featureID=1586&type=3&iaid=133 or by e-mail: disparitiesresearch@rwjf.org.

